# SARS-CoV-2 spike protein induces lung endothelial cell dysfunction and thrombo-inflammation depending on the C3a/C3a receptor signalling

**DOI:** 10.1038/s41598-023-38382-5

**Published:** 2023-07-14

**Authors:** Luca Perico, Marina Morigi, Anna Pezzotta, Monica Locatelli, Barbara Imberti, Daniela Corna, Domenico Cerullo, Ariela Benigni, Giuseppe Remuzzi

**Affiliations:** Istituto di Ricerche Farmacologiche Mario Negri IRCCS, Bergamo, Italy

**Keywords:** Microbiology, Virology, Cell biology, Mechanisms of disease

## Abstract

The spike protein of Severe Acute Respiratory Syndrome Coronavirus 2 (SARS-CoV-2) can interact with endothelial cells. However, no studies demonstrated the direct effect of the spike protein subunit 1 (S1) in inducing lung vascular damage and the potential mechanisms contributing to lung injury. Here, we found that S1 injection in mice transgenic for human angiotensin converting enzyme 2 (ACE2) induced early loss of lung endothelial thromboresistance at 3 days, as revealed by thrombomodulin loss and von Willebrand factor (vWF) increase. In parallel, vascular and epithelial C3 deposits and enhanced C3a receptor (C3aR) expression were observed. These changes preceded diffuse alveolar damage and lung vascular fibrin(ogen)/platelets aggregates at 7 days, as well as inflammatory cell recruitment and fibrosis. Treatment with C3aR antagonist (C3aRa) inhibited lung C3 accumulation and C3a/C3aR activation, limiting vascular thrombo-inflammation and fibrosis. Our study demonstrates that S1 triggers vascular dysfunction and activates complement system, instrumental to lung thrombo-inflammatory injury. By extension, our data indicate C3aRa as a valuable therapeutic strategy to limit S1-dependent lung pathology.

## Introduction

The novel coronavirus, called Severe Acute Respiratory Syndrome Coronavirus 2 (SARS-CoV-2), is the causative agent of the Coronavirus Disease 2019 (COVID-19), which emerged as a pandemic disease claiming, as of April, 2023, almost 7 million lives worldwide^1^.

The cell target of SARS-CoV-2 infection are epithelial cells in the upper airways^2,3^. In about 20% of patients, SARS-CoV-2 infection expands to the distal lung and rapidly deteriorates to a severe illness, characterised by bilateral interstitial pneumonia, acute respiratory distress syndrome, and multi-organ damage with a high fatality rate^4^. Vascular abnormalities across different organs have been identified as the most frequently reported complications^5–8^. Histologic analysis of pulmonary vessels showed a high frequency of widespread thrombosis with microangiopathy in alveolar capillary of COVID-19 patients^7,9^. Although endothelial cell dysfunction is a prominent feature of severe COVID-19, the pathogenetic mechanisms through which SARS-CoV-2 induces endotheliopathy still remain elusive.

Endothelial cells express the SARS-CoV-2 entry machinery, including angiotensin-converting enzyme 2 (ACE2), as the main viral receptor widely distributed in many tissues^10–12^. Furthermore, several additional endothelial surface receptors for SARS-CoV-2 have been identified, including the transmembrane glycoprotein CD147 and neuropilin-1 (NRP1)^13,14^. Interaction between SARS-CoV-2 and endothelial cells is likely to occur in the lungs when adjacent pulmonary epithelium is damaged via apoptotic processes during viral infection, as well as in peripheral organs when a high viral load is present in the blood^15–17^.

Most of the available data indicate that SARS-CoV-2 cannot replicate in endothelial cells^15^, implying that the endothelial cell dysfunction is the result of the intracellular signaling activated by the binding of the spike protein to ACE2^18^. This interaction modifies endothelial cell phenotype by enhancing the production of cytokines and reactive oxygen species, as well as impairing cell permeability and metabolic functions^19–23^. Our group showed that the subunit 1 (S1) of the spike protein, which contains the receptor binding domain (RBD) for ACE2, is sufficient per se to alter the microvascular endothelial cell phenotype in vitro, propagating microvascular inflammatory and thrombogenic processes^24^. S1 promotes leukocyte adhesion and thrombus formation under flow condition on cultured human endothelial cells by enhancing the surface expression of pro-inflammatory adhesive molecules for leukocyte, intercellular adhesion molecule 1 (ICAM-1) and P-selectin, and the pro-thrombogenic protein von Willebrand factor (vWF), as well as inducing the loss of thrombomodulin (TM)^24^. The detrimental effects of S1 are amplified by the activation of the complement system, as the C3 and C5-b9 deposition on endothelial cells upon exposure to S1 and the formation of platelet aggregates were inhibited by the C3 inhibitor compstatin, as well as by C3a and C5a inhibitors^24^.

In the clinical setting, the activation of complement system has been shown to associate with vascular injury and thrombosis in severe COVID-19 patients^25–27^. Among the most compelling findings is that anaphylatoxins C3a and C5a, generated during the proteolytic cleavage of C3 and C5, as well as C5b-9, correlated with disease severity and mortality^25,26^. Several case series studies suggested a potential benefit of different complement inhibitors^25,26^. Clinical trials are now beginning to substantiate these findings, although with mixed results regarding efficacy and risk profile^25,26^. Hence, a better understanding of the pathogenic role of complement proteins in animal models is needed to optimize targeted therapies.

Here, the aim of the present study is to characterize the pathogenic effects of SARS-CoV2-derived S1 on the lung vasculature in mice transgenic for human ACE2. In this model, we also explore the ability of S1 to activate the complement system, which may contribute to lung damage. Finally, we assess the therapeutic potential of a C3a receptor antagonist treatment in mice with COVID-19-like lung injury.

## Results

### Injection of SARS-CoV2-derived S1 induces lung tissue injury and fibrosis in hACE2-KI mice

**Figure 1:**
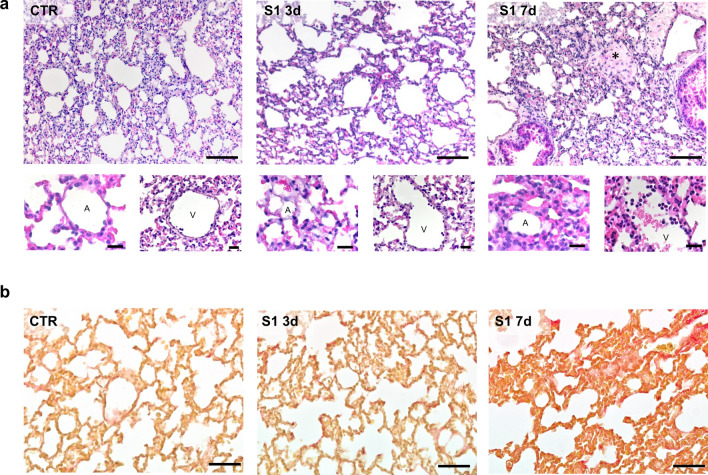
S1 injection induces tissue injury and fibrosis in the lung of hACE2-KI mice. (**a**) Histologic analysis of lung structures stained with hematoxylin–eosin in control (CTR, n = 4) or S1-injected mice at 3 (n = 3) and 7d (n = 6). Scale bars: 100 µm. Lung tissues from CTR mice show thin lining of pneumocyte type I and II surrounding the alveoli (A). At 3d, lung tissues of mice exposed to S1 exhibit an almost intact alveolar architecture, while at 7d lung tissue displays alveolar septal thickening, hypercellularity and collagen deposition in the parenchyma (asterisk). At 7d after S1 injection, an increase in inflammatory cells within vessels (V) is evident. The lower images show the structure of alveoli (A) and blood vessels (V) in all experimental groups at a higher magnification. Scale bars: 20 µm. (**b**) Representative images of lung fibrosis assessed by Sirius red staining in control or S1-injected mice at 3 and 7d. Scale bars: 50 µm.

In order to maximize the binding activity of the S1 subunit of the SARS-CoV-2 spike protein, we elected to use transgenic mice with the endogenous mouse *Ace2* sequences replaced by the human ACE2 cDNA (hACE2-KI). These mice were intravenously injected with 35 μg of recombinant S1 and sacrificed at 3 and 7 days. Analysis of hematoxylin and eosin-stained lung sections from mice injected with S1 revealed an almost intact alveolar architecture at 3 days, similar to those observed in control animals (Fig. 1a). Conversely, histopathological examination of lung tissue at 7 days showed diffuse alveolar damage, as revealed by alveolar septal thickening, associated with lung tissue hypercellularity caused by a progressive infiltration of inflammatory cells within the alveolar parenchyma and in blood vessels, perivascular sites and also spreading to larger interstitial areas (Fig. 1a). Concomitantly, alveolar wall thickening and the presence of inflammatory cells were associated with fibrotic tissue accumulation in the lung parenchyma (Fig. 1a,b). To fully appreciate the pro-fibrotic effect of S1 in the lungs, Sirius red staining was performed. As shown in Fig. 1b, a significant increase in interstitial and intra-alveolar collagen deposition was found in the lung of mice at 7 days, while at 3 days collagen deposition was similar to that of untreated mice.

### Injection of SARS-CoV2-derived S1 triggers activation of complement system and C3a/C3aR axis in the lung tissue of hACE2-KI mice

**Figure 2:**
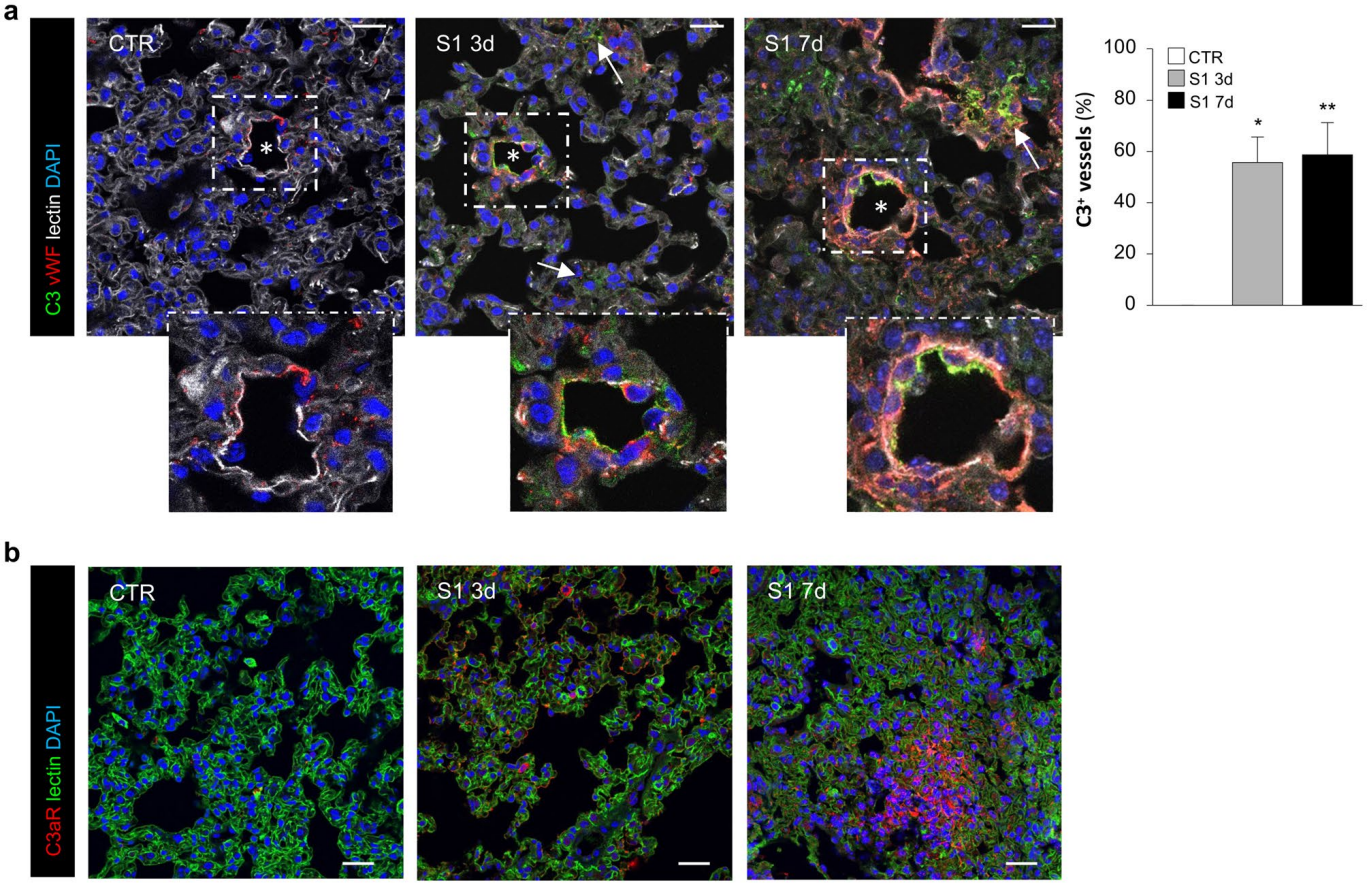
S1 injection induces endothelial C3 deposits and increases C3aR expression in lung tissue of hACE2-KI mice. (**a**) Representative images of C3 deposits (green) in lung vessels (asterisk) positive for vWF (red) and in lung parenchyma (arrows) in CTR (n = 4) or S1-injected mice at 3 (n = 3) and 7d (n = 6). Insets display vascular C3 deposition. Endothelial C3 deposits are quantified and expressed as % of C3 positive vessels. Lung structures and nuclei are counterstained with wheat germ agglutinin (WGA) lectin (white) and DAPI (blue), respectively. Scale bars: 20 µm. Results are presented as mean ± SEM. *P < 0.05 and **P < 0.01 versus CTR. (**b**) Representative images of C3aR staining (red) in lung vessels and in lung parenchyma in CTR (n = 4), S1-injected mice at 3d (n = 3), and 7d (n = 6). Lung structures and nuclei are counterstained with WGA lectin (green) and DAPI (blu), respectively. Scale bars: 20 µm.

To investigate the detrimental role of complement activation in the development of lung vascular injury in hACE2-KI mice injected with S1, we first evaluated the presence of pulmonary C3 deposits. A significantly enhanced staining of C3 was found early at 3 days following S1 injection, which persisted at 7 days (Fig. 2a), both in focal area of parenchyma (arrows) and in vessels (asterisks). No or rare signs of C3 staining were found in control animals (Fig. 2a). In order to evaluate the extent of C3 deposits in the lung vasculature, we quantified the number of vWF-labeled lung vessels positive for the C3. In this setting, we observed a significant increase in the percentage of vessels with C3 deposits starting at 3 days that remained consistently high at 7 days (Fig. 2a). Similar data were obtained when the area of C3 staining *per* vessel was quantified (Supplementary Figure 1a). In this context, the enhanced C3 deposits was paralleled by a remarkable increase in the expression of vWF (Fig. 2a). Then, we analyzed the expression of CD46, a surface complement regulator known to inhibit the formation of C3 convertase by the alternative, classic, and lectin pathways^28^. We observed a dramatic reduction in CD46 protein expression in alveolar epithelial and endothelial cells both at 3 and 7 days after S1 injection (Supplementary Figure 1b).

Since the activation of complement system and exuberant C3 deposits lead to increased C3a generation^25,26^, we further investigated the possible detrimental activity of C3a/C3a receptor (C3aR) axis in S1-induced lung tissues injury. For this purpose, the expression of the specific C3aR at different time intervals were studied in the distinct lung compartments. In control hACE2-KI mice, C3aR expression was not expressed in alveolar epithelial cells and lung vessels (Fig. 2b), while it mainly localized in bronchiolar epithelial cells (Supplementary Figure 2a, asterisks). Conversely, mice injected with S1 exhibited a progressive increase in the C3aR expression in vessels and lung parenchyma starting at 3 days, with the maximum increased observed at 7 days (Fig. 2b). These findings indicate that the activation of complement system in the lung, in terms of abnormal C3 deposits, reduced CD46 expression, and activation of C3a/C3aR signalling are observed early at 3 days, preceding temporally lung injury and fibrosis in hACE2-KI mice.

### Injection of SARS-CoV2-derived S1 induces endothelial cell injury in the lung promoting vascular thrombo-inflammation and fibrosis

**Figure 3:**
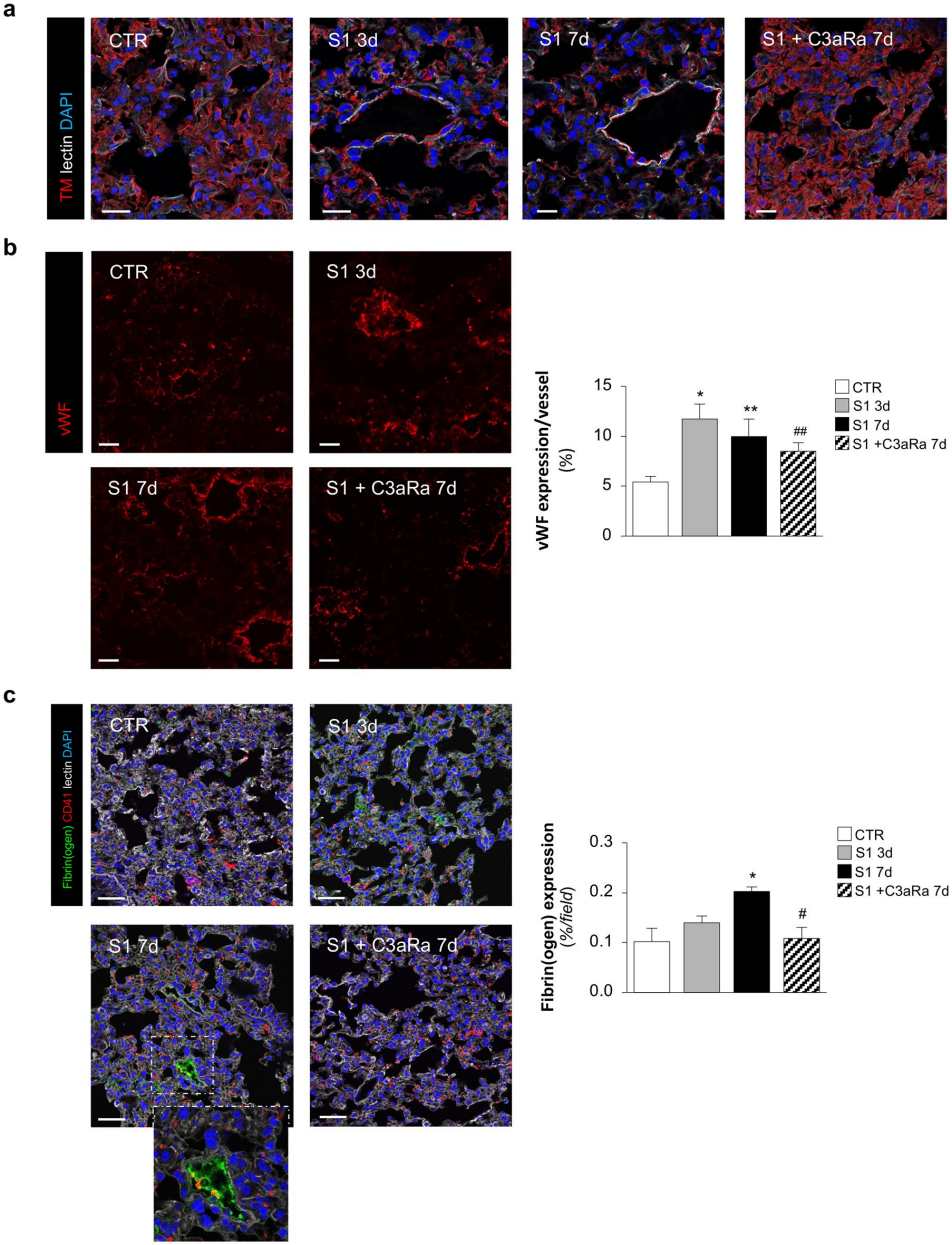
C3aR antagonist reduces endothelial cell injury in lung tissue of hACE2-KI mice injected with S1. (**a**) Representative images of thrombomodulin (TM, red) staining in CTR (n = 4), S1-injected mice at 3d (n = 3), and 7d treated or not with C3aRa (SB290157) 5 mg/kg daily (n = 6 *per* group). Lung structures and nuclei are counterstained with WGA lectin (white) and DAPI (blue), respectively. Scale bars: 20 µm. (**b**) Representative images and quantification of von Willebrand (vWF) staining (red) in CTR (n = 4), S1-injected mice at 3d (n = 3), and 7d treated or not with C3aRa (n = 6 *per* group). Scale bars: 20 µm. Results are presented as mean ± SEM. *P < 0.05 and **P < 0.01 *vs* CTR; ^##^*P* < 0.01 *vs* S1 7d. (**c**) Representative images and quantification of fibrin(ogen) (green) in co-staining with CD41 (red) in CTR (n = 4), S1-injected mice at 3d (n = 3), and 7d treated or not with C3aRa (n = 6 *per* group). Lung structures and nuclei are counterstained with WGA lectin (white) and DAPI (blue), respectively. Scale bars: 20 µm. Results are presented as mean ± SEM. *P < 0.05 versus CTR; ^#^*P* < 0.05 versus S1 7d.

Recent in vitro data by our group demonstrate that S1 is sufficient to alter pulmonary endothelial cell phenotype, leading to vascular loss of anti-thrombogenic properties and induction of inflammatory cell response^24^. To establish whether S1 infusion in hACE2-KI mice induced lung vascular cell damage, we evaluated a series of markers known to be involved in thrombo-inflammatory processes. First, we studied the expression of thrombomodulin (TM), a glycoprotein known to confer cyto-protection, anti-inflammatory and thromboresistance properties to endothelial cells^24^. We observed that injection of S1 cause a robust reduction in TM expression already at 3 days in respect to control animals that showed a marked constitutive staining of TM (Fig. 3a). This finding was accompanied by a significant increase in the endothelial expression of the pro-thrombotic protein vWF in response to S1, as compared to that observed in endothelial cells of control mice (Fig. 3b). The increased positivity of endothelial cells to vWF started at 3 days from S1 infusion, possibly reflecting the endothelial activation and exocytosis of the protein. The activation of the endothelial pro-thrombotic state was sustained also by data that deposits of fibrin(ogen) markedly increased and co-stained with CD41 positive platelets in the vascular compartment in response to S1 at 7 days (Fig. 3c, inset). No staining of fibrin(ogen) was observed in control mice (Fig. 3c).

**Figure 4:**
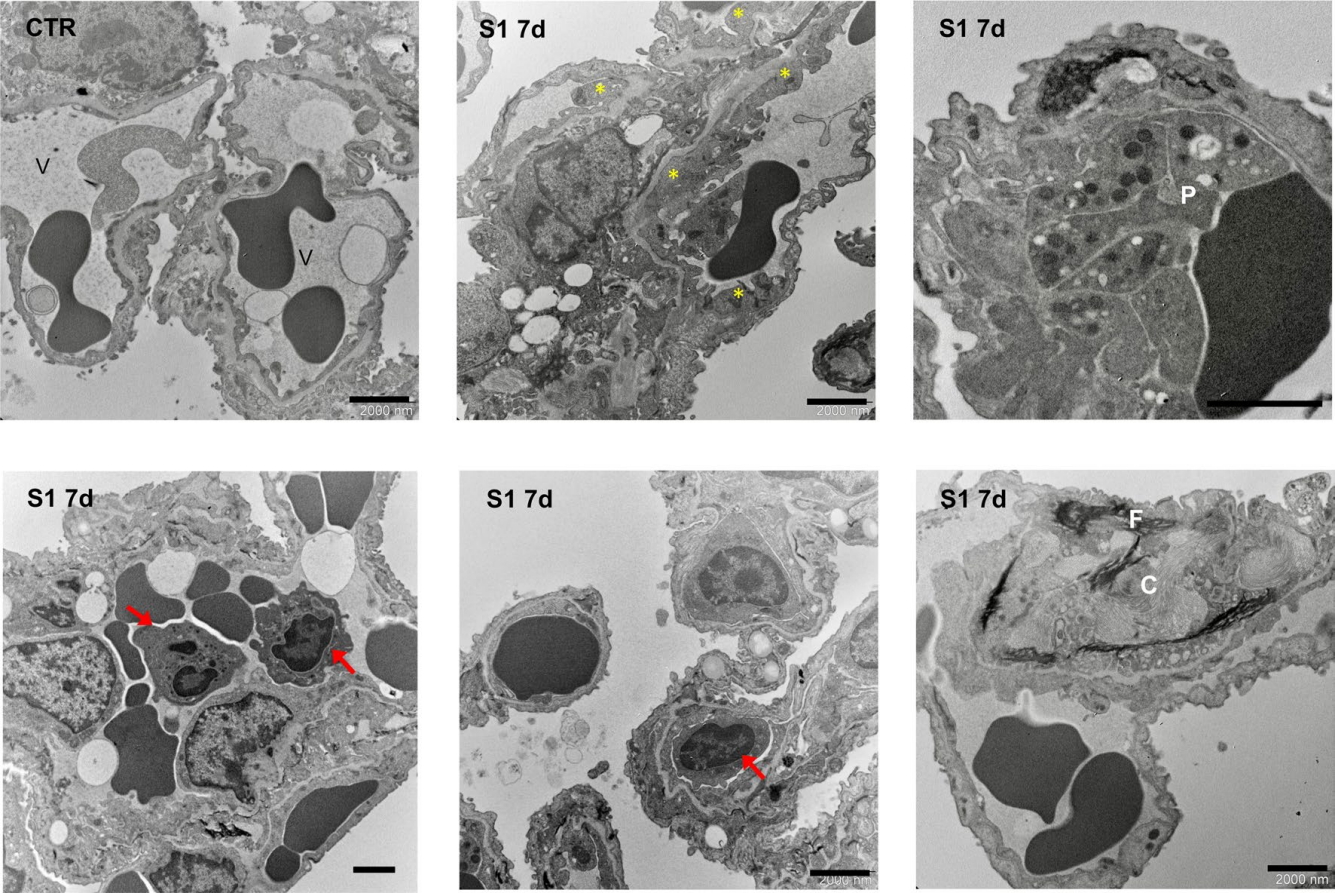
S1 injection induces endothelial ultrastructural changes in lung tissue of hACE2-KI mice. Ultrastructural analysis of lung alveolar tissue from CTR and S1-injected mice at 7d. In CTR mice, thin vascular endothelial cells form the inner lining of alveolar capillary (V). Mice injected with S1 at 7d show areas of thickening of endothelial cells (yellow asterisks), associated with activated platelets (P), inflammatory cell accumulation (red arrows), including neutrophils and monocytes, and material with fibrillar aspect (F) and collagen (C) deposition. Scale bars: 2000 nm.

All these results are consistent with data obtained by transmission electron microscopy analysis of lung tissues of hACE2-KI mice at 7 days from S1 injection. While in control mice the alveolar blood capillaries are lined with thin non-fenestrated endothelial cells, areas of endothelial swelling were observed in the pulmonary microcirculation (Fig. 4, asterisks) and arterioles (Supplementary Figure 3) in S1-injected mice. In addition, activated platelets were focally found adhering to damaged endothelial cells, while no signs of platelet adhesion were observed in control mice (Fig. 4). Lung ultrastructural examination also revealed the presence of inflammatory cells infiltrating the injured vessels, consisting of both neutrophils and macrophages at 7 days from S1 injection (Fig. 4, arrows). The accumulation of fibrotic tissue with fibrillar aspects and collagen deposition was observed predominantly in interstitial area and alveolar septa (Fig. 4).

### C3aR blockade limits endothelial cell injury in the lung induced by SARS-CoV2-derived S1

To demonstrate that complement activation is crucial in potentiating the S1-induced endothelial injury in the lung, we studied the effects of daily administration of C3a receptor antagonist (C3aRa) SB290157 starting 8 h after S1 injection in hACE2-KI mice. We found a significant reduction of the endothelial C3 deposits induced by S1 at 7 days (Supplementary Figure 1a), accompanied by restoration of CD46 expression (Supplementary Figure 1b). In parallel, we found a decreased expression of C3aR in the lung tissue (Supplementary Figure 2b), confirming the ability of SB290157 to limit the activation of complement and C3a/C3aR signaling. Importantly, blockade of C3aR resulted in a significant limitation of vascular damage of lung tissue, as revealed by the restoration of TM expression (Fig. 3a) and reduction of vWF staining along vessel wall at 7 days (Fig. 3b). A similar trend was observed for endothelial fibrin(ogen) deposition that was restored to control levels by the administration of C3aRa (Fig. 3c).

**Figure 5:**
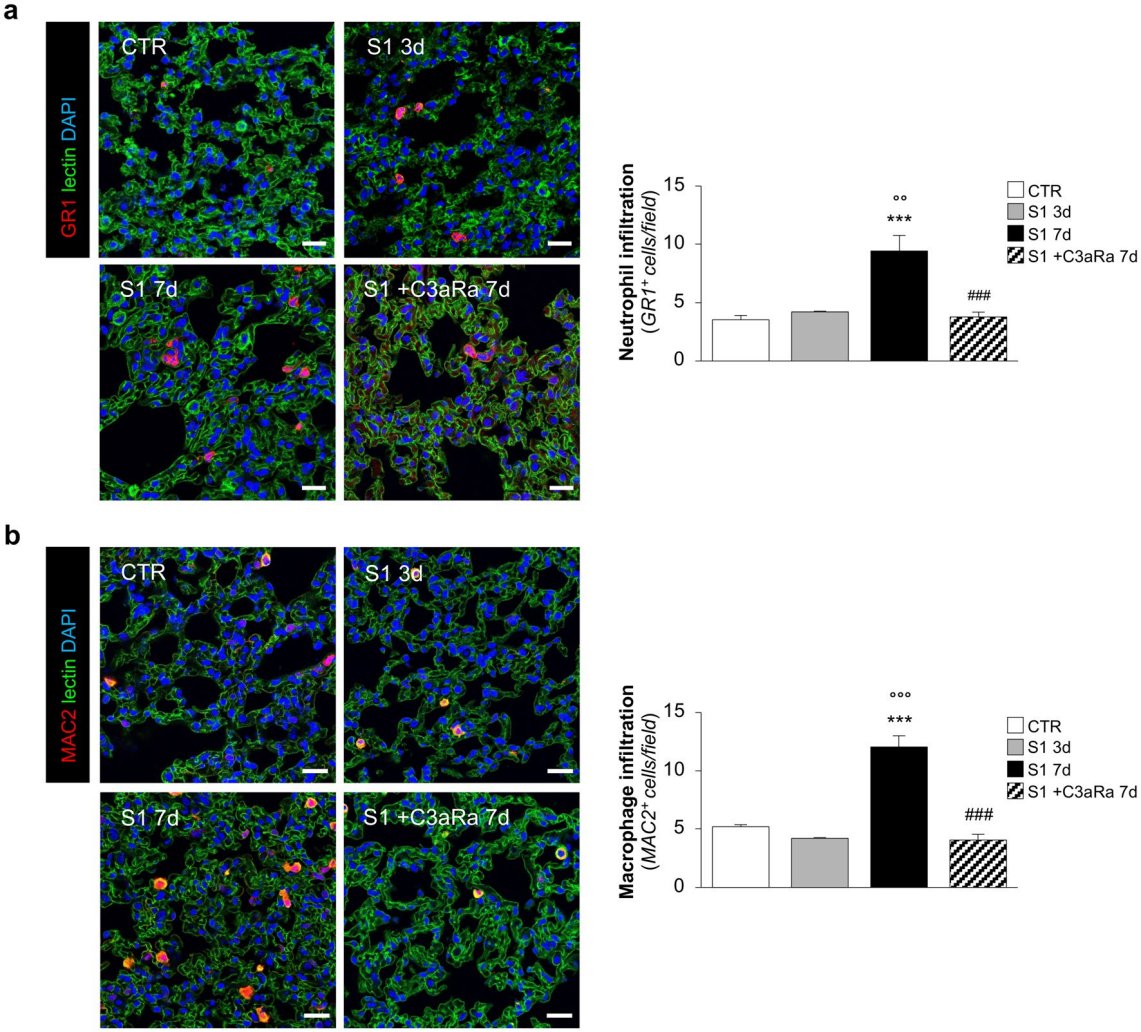
C3aR antagonist inhibits inflammatory cell infiltration in lung tissue of hACE2-KI mice injected with S1. (**a**) Representative images and quantification of GR1^+^ neutrophils (red) in CTR (n = 4), S1-injected mice at 3d (n = 3), and 7d treated (n = 6) or not (n = 5) with C3aRa. Results are presented as mean ± SEM. ***P < 0.001 versus CTR; °°*P* < 0.01 versus S1 3d; ^###^*P* < 0.001 versus S1 7d. (**b**) Representative images and quantification of MAC2^+^ macrophages (red) in CTR (n = 4), S1-injected mice at 3d (n = 3), and 7d treated or not with C3aRa (n = 6 *per* group). Lung structures and nuclei are counterstained with WGA lectin (green) and DAPI (blue), respectively. Scale bars: 20 µm. Results are presented as mean ± SEM. ***P < 0.001 versus CTR; °°°*P* < 0.001 versus S1 3d; ^###^*P* < 0.001 versus S1 7d.

Given the profound effect of C3a as a potent chemotactic factor for the recruitment of inflammatory cells^29^, we sought to investigate the effect of C3aRa on inflammatory cell infiltration in the lungs of S1-treated hACE2-KI mice. Immunofluorescence analysis and quantification of neutrophils and macrophages was performed by analysis of the specific markers GR1 and MAC2, respectively. In lung tissue at 7 days following S1 injection a significant accumulation of neutrophils (GR1^+^) and macrophages (MAC2^+^) was found, compared to untreated mice (Fig. 5a,b). Finding that inflammatory cell infiltration was not evident at 3 days (Fig. 5a,b) suggested that the early activation of the complement system was instrumental for inflammatory cell recruitment into the lungs. That this is the case was confirmed by finding that C3aRa completely blocked both pulmonary neutrophil and macrophage infiltration at 7 days (Fig. 5a,b). Of note, we found that S1 injection induced the formation of scattered neutrophil extracellular traps (NETs) within lung tissues, as revealed by decondensation of DNA resulting in a positive double staining for two bactericidal proteins myeloperoxidase (MPO, green) and neutrophil elastase (NE, red), which was not evident in untreated mice (Supplementary Figure 4). Treatment with C3aRa was effective in inhibiting NET formation induced by the S1 protein (Supplementary Figure 4).

### C3aR blockade limits lung tissue injury and fibrosis induced by SARS-CoV2-derived S1

**Figure 6:**
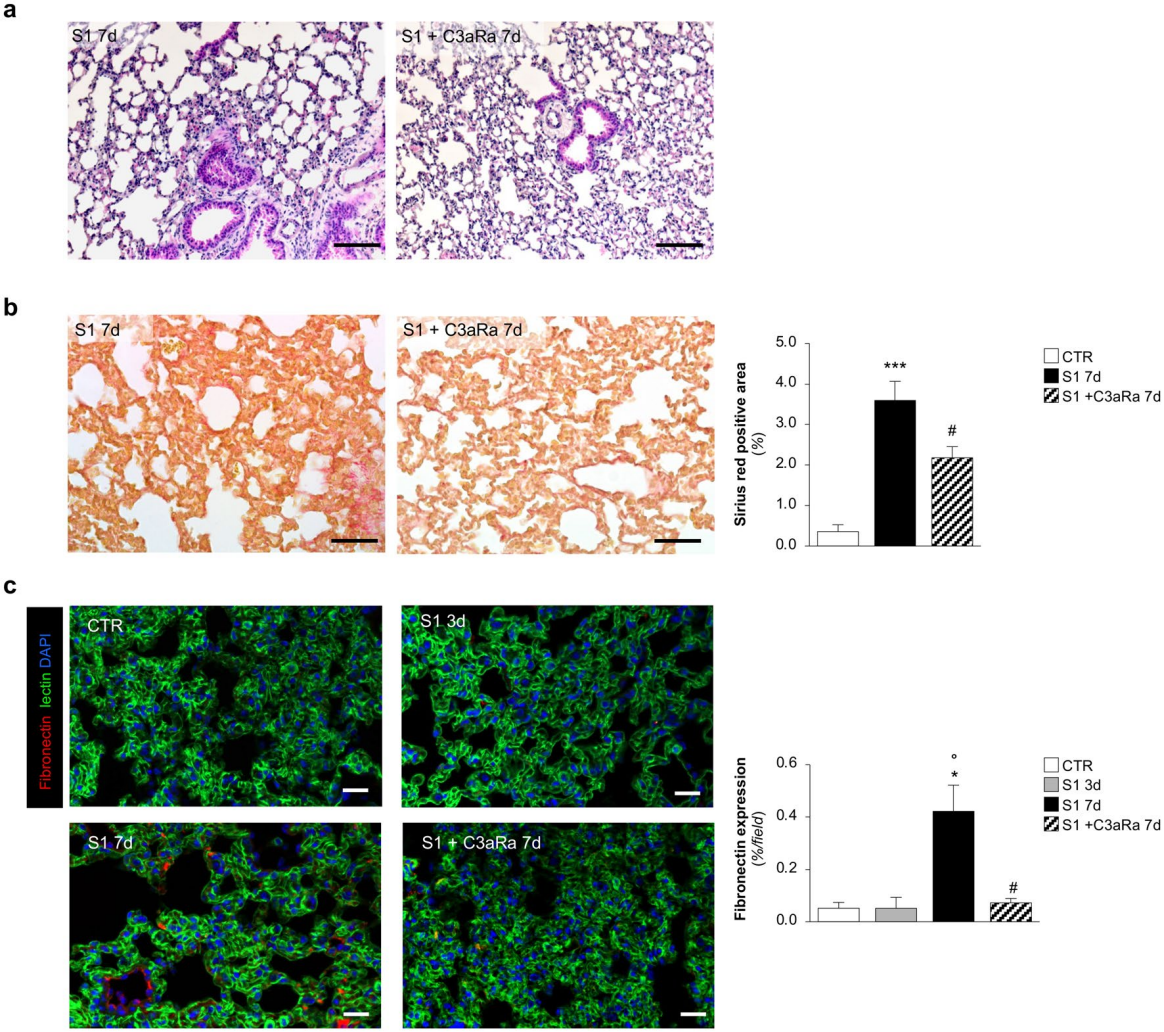
C3aR antagonist limits lung tissue injury and fibrosis in hACE2 mice injected with S1. (**a**) Representative images of hematoxylin/eosin staining in mice injected with S1 and treated or not with C3aRa at 7d (n = 6 *per* group). Scale bars: 100 µm. (**b**) Representative images and quantification of lung fibrosis assessed by Sirius red staining in S1-injected mice at 7d treated or not with C3aRa (n = 6 *per* group). Scale bars: 50 µm. Results are presented as mean ± SEM. ***P < 0.001 *vs* CTR; ^#^*P* < 0.05 versus S1 7d. (**c**) Representative images and quantification of fibronectin staining (red) in CTR (n = 3), S1-injected mice at 3d (n = 3), and 7d treated or not with C3aRa (n = 6 *per* group). Lung structures and nuclei are counterstained with WGA lectin (green) and DAPI (blue), respectively. Scale bars: 20 µm. Results are presented as mean ± SEM. *P < 0.05 versus CTR; °*P* < 0.05 versus 3d; ^#^*P* < 0.05 versus 7d.

To analyze the full spectrum of the therapeutic potential of complement inhibition, we investigated the effect of C3aRa on lung histologic changes and fibrosis. As shown in Fig. 6a, treatment with C3aRa was sufficient to preserve alveolar architecture in S1-treated hACE2-KI mice at 7 days, as revealed by reduction of alveolar structural alteration, including septal thickening, when compared with S1-treated mice given saline. Similarly, treatment with C3aRa significantly limit fibrotic tissue accumulation in the lung parenchyma of S1-treated hACE2-KI mice at 7 days (Fig. 6b). The ability of C3aRa to reduce the fibrotic processes in the lung of S1-treated hACE2-KI mice was further confirmed by the immunofluorescence analysis of fibronectin staining in the lungs. hACE2-KI mice showed increased vascular deposits of fibronectin at 7 days that were completely inhibited by blockade of complement activation with the C3aR antagonist (Fig. 6c).

## Discussion

Endothelial cell injury is recognized as important driver of the severe COVID-19 pathogenesis^6,26^, although the detailed mechanism underlying its occurrence following SARS-CoV-2 infection is still poorly understood. In the present study, we show that systemic delivery of the SARS-CoV-2-derived spike protein S1 elicits per se a progressive lung injury in mice, characterized by early endothelial cell dysfunction, leading to subsequent thrombo-inflammatory and fibrotic damage.

To the best of our knowledge, only few studies assessed the detrimental effects of S1 on the lung in vivo^30,31^. Specifically, S1 instillation significantly induced lung injury in transgenic mice with selective expression of human ACE2 under the keratin 18 promoter in the epithelia (K18-hACE2), ruling out the possibility to dissect the effect of S1 on the lung vasculature^30,31^. To overcome this limitation, we elected to use transgenic mice expressing human ACE2 under the murine *Ace2* promoter (hACE2-KI), that engender human ACE2 in the endothelia of whole body. In these mice, we systemically delivered S1 in order to mimic the high burden of circulating spike protein found in severe COVID-19 patients and correlating with disease severity^32^. That systemic delivery of viral antigens is effective in targeting the lungs was provided by two independent studies showing that, compared to other organs, the lungs had the highest full-length spike and S1 uptake^33,34^. Of note, the critical role of ACE2 in pulmonary tropism of the viral antigens was offered by data showing that co-administration of the full-length spike or S1 with recombinant ACE2 or anti-ACE2 antibody suppressed their uptake in the lungs^33,34^. Based on these data, our model could represent a suitable experimental tool to study the impact of S1 on endothelial function. Indeed, one of the most striking and early pathologic hallmarks following S1 injection was the prominent vascular injury in the lung at 3 days after S1 injection, characterized by loss of TM, as well as increased vascular vWF. Externalization of vWF is likely the result of exocytotic activity from Weibel-Palade bodies, although an increased protein synthesis cannot be excluded. All the changes in the vascular compartment were prodromal to the full blow lung injury characterized by a strong, diffuse alveolar damage, vascular fibrin(ogen) deposition and platelet adhesion, as well as inflammatory cell recruitment and fibrosis at 7 days.

That changes in the lung observed in our model mimic lung injury reported with full competent virus is confirmed by a study in K18-hACE2 mice, in which viral localization in lung capillary after intranasal injection of SARS-CoV-2 resulted in endothelial activation and dysfunction, severe interstitial lung inflammation, hemorrhage, as well as perivascular inflammation^35^. In agreement with our data, intratracheal instillation of S1 in K18-hACE2 was sufficient to induce a COVID-19-like acute lung injury and endothelial cell dysfunction^31^. Similar detrimental effects on lung vascular injury were observed when a non-infectious pseudovirus expressing the spike protein on its surface, without additional SARS-CoV-2 components, were instilled in Syrian hamster^20^. Lastly, in agreement with data in our model, a recent study showed a pro-fibrotic effect of an aerosol-delivered pseudovirus expressing the spike protein in the lung of mice with doxorubicin-induced hACE2^36^.

A further prominent finding of our study is the observation that early activation of the complement system occurred concomitant with endothelial cell dysfunction, as revealed by detection of C3 deposits in both the endothelial and epithelial cell compartment of the lungs, as well as increased expression of C3aR. Our data are consistent with the few available in vivo studies showing that complement activation was associated with endotheliitis and vasculitis in response to SARS-CoV-2 or S1 exposure^37–39^. Having demonstrated that complement activation is one of the earlies features that preceded thrombo-inflammatory and fibrotic processes in our experimental model, gave us the unprecedented opportunity to dissect the actual role of the complement system in the pathophysiology of S1-induced endothelial cell dysfunction and the subsequent lung injury. The proof-of-concept that complement proteins are active players in triggering lung injury in response to S1, rests on the present data that early treatment with the C3aR antagonist SB290157 is able to inhibit lung C3 deposits and C3a/C3aR axis activation, limiting the progression of lung vascular dysfunction and the consequent development of thrombo-inflammatory injury and fibrosis. These findings are consistent with the available clinical data indicating that early intervention with complement inhibitors is preferred in severe COVID-19 patients, while late treatments are often ineffective^26^.

Mechanistically, finding that C3aR antagonist blocks endothelial C3 deposition can be explained by previous studies showing that C3a favors the exocytosis of Weibel-Palade bodies^24,40^, with the consequent endothelial surface mobilization of the prothrombotic vWF, P-selectin, and tissue plasminogen activator (t-PA). P-selectin is a well-known ligand for C3 through high-affinity binding^40^. Our finding that C3aR antagonist limits lung vascular expression of vWF, possibly implies the concomitant reduction of P-Selectin exocytosis and the consequent C3 binding on endothelial cells. Similarly, the potential ability of C3aR antagonist to halt t-PA exocytosis may result in a decreased cleavage of TM, which we actually found preserved by C3aR antagonist following S1 exposure. TM limits complement activation and deposition on endothelial cell surface, as well as impedes endothelial-platelet interaction and thrombus-formation^41^. On the top of that, the ability of C3aR antagonist to preserve the expression of CD46, a C3 convertase inhibitor, adds a further degree in the complex regulation of C3 and generation of C3a in lung endothelial cells following S1 exposure.

Considering that C3a is traditionally described as pro-inflammatory modulator^42^, next we sought to investigate the potential of targeting C3aR on lung neutrophil and macrophage infiltration in response to S1-injection. Our findings clearly showed a reduction of S1-induced inflammatory cell recruitment in the lung parenchyma and vessels upon C3aRa treatment. Of particular interest, C3aR blockade was also effective in limiting the formation of neutrophil extracellular traps (NETs), consistent with previous in vitro study^43^. C3a/C3aR axis is a potent inducer of NETosis in neutrophils, contributing to blood clot formation^44^. Upregulation of C3aR on neutrophils and extensive spontaneous NET formation have been found to correlate with endothelial dysfunction in children with multisystem inflammatory syndrome (MIS-C)^45,46^, as well as in severe COVID-19 patients^47,48^.

An additional layer of protection of C3aR antagonist was also provided by its ability to arrest the progression of fibrosis, as revealed by local reduction of extracellular matrix accumulation in the lung. These data are consistent with a previous study showing that blockade of C3a/C3aR signaling is critical to the pathogenesis of pulmonary fibrosis by suppressing TGFβ signaling activity^49^.

Collectively, our data provide novel mechanistic insights into the complex interplay between endothelial dysfunction and complement activation as the underlying cause of lung injury induced by S1 protein and suggests C3a and C3aR as potential promising therapeutic targets for the clinical management of COVID-19 patients.

## Methods

### Ethical statement

All procedures involving animals were performed in accordance with institutional guidelines in compliance with national (D.L.n.26, March 4, 2014), and international laws and policies (directive 2010/63/EU on the protection of animals used for scientific purposes). This study was approved by the Institutional Animal Care and Use Committees of Istituto di Ricerche Farmacologiche Mario Negri IRCCS.

### Animal experiments

Male hemizygous B6.129S2(Cg)-Ace2^tm1(ACE2)Dwnt^/J (hACE2-KI) mice (strain #:035000; The Jackson Laboratory) were maintained in a specific pathogen-free facility at a constant temperature with a 12:12-h light/dark cycle and free access to a diet and water. At 12 weeks of age, hACE2-KI mice were injected intravenously with 35 μg SARS-CoV-2 spike protein S1 produced in HEK cells (130–127-854, Miltenyi). This dose was chosen on the basis of preliminary experiments with different S1 concentrations, starting from previously reported data^50^. The previously published lower S1 dose^50^ induced a minimal lung damage in our experimental setting.

Mice injected with 35 μg S1 were sacrificed at 3 day (n = 3) and 7 days (n = 6). An additional group of hACE2-KI mice (n = 6) received the C3aRa SB290157 (1140525–25-2, Cayman Chemical) at a dose of 2.5 mg/kg by intraperitoneal injection twice a day, starting 8 h after S1 injection and sacrificed at 7 days. Animals receiving S1 alone were injected with the appropriate vehicle^51^. Untreated hACE2-KI mice were used as control group (CTR, n = 4). Mice were euthanized through CO_2_ and lungs processed for subsequent analyses. No inclusion or exclusion parameters were used. Investigators were blinded to treatments. This study was carried out in compliance with the ARRIVE guidelines^52^.

### Lung histology and fibrosis

Lung samples were fixed in formalin. Paraffin-embedded sections (4 µm) were stained with hematoxylin–eosin and assessed by light microscopy. Lung tissue fibrosis was examined in paraffin-embedded sections stained with Picro Sirius red. The percentage of total area positive for Sirius red staining was quantitated in 15 to 20 fields (HPF, × 20) *per* lung using Fiji ImageJ software (http://imagej.net/Fiji). Digitized images were dichotomized using a threshold for Sirius red staining, and the values were expressed as the percentage of staining per total field area. Sections were examined using ApoTome Axio Imager Z2 (Zeiss, Jena, Germany) and were analyzed by the same pathologist who was blinded to the experimental conditions.

### Immunofluorescence analysis

Optimum Cutting Temperature (OCT)-frozen lung sections (4-μm thick) were fixed with cold acetone or fixed in periodate-lysine-paraformaldehyde (PLP) incubated with 1% BSA to block nonspecific sites, as previously described^53^. The sections were then incubated with the following primary antibodies: rabbit anti-C3aR antibody (1:100; LS-C382362, Lifespan BioSciences Inc.); rabbit anti-vWF (1:3000; A0082, Dako); rabbit anti-NE (1:100; GTX66150, GeneTex); goat anti-MPO (1:66; AF3667, R&D System); goat anti-TM (1:200; MAB3894, R&D System); rat anti-CD41 (1:100; ab33661, Abcam); rat anti-GR1 antibody that recognizes the lymphocyte antigen 6 complex locus G6D (Ly6G; 1:50; RM3000, Caltag); rat anti-macrophage antigen 2 (MAC2; 1:200; CL8942AP, Cederlane) followed by the corresponding Cy3-conjugated secondary antibodies (Jackson ImmunoResearch Laboratories), as appropriate. For double staining experiments, FITC-conjugated goat anti-mouse C3 (1:200; 55,500, Cappel) and FITC-conjugated Fibrin(ogen) (1:50; F0111, Dako) were used. Negative controls were obtained by omitting the primary antibody on adjacent sections. Lung structure were counterstained with FITC- or Alexa Fluor 633-wheat germ agglutinin (WGA) lectin (FL-1021 Vector Laboratories, or W21404 Invitrogen), as appropriate. Nuclei were counterstained with DAPI. Samples were examined under confocal inverted laser microscopy (Leica TCS SP8, Leica Microsystems, Wetzlar, Germany).

The expression of fibrin(ogen) and fibronectin in the lung were quantified with ImageJ. Digitized images were binarized using a threshold, the values were expressed as a percentage of area occupied by staining *per* total area of the acquired field (n = 15 randomly selected HPF).

Expression of vWF in the lung was quantified and expressed as vWF positive area *per* vessel in an average of 15 fields.

For the expression of C3 in lung vessels, the number of C3-positive vessels, identified by the specific endothelial marker vWF, was counted in an average of 15 fields and expressed as the % on total vessels *per* field. Moreover, lung C3 deposits expressed as C3 positive area *per* vessel were also quantified in an average of 15 fields.

For quantification of GR1^+^ or MAC2^+^ cells were counted in an average of 15 fields and expressed as the average number of cells *per* field.

### Immunohistochemistry analysis

The expression of CD46 was assessed by using the immunoperoxidase method. Formalin-fixed, 4-μm paraffin-embedded kidney sections were incubated for 5 min with Peroxidazed 1 (PX968, Biocare Medical, Pacheco, CA, USA) to quench endogenous peroxidase, after antigen retrieval in Rodent Decloaker (RD913, Biocare Medical). After blocking for 10 min with Rodent Block M (RBM961G, Biocare Medical), sections were incubated with antibody anti-CD46 (1:200; PA5-95,788, ThermoFisher) followed by rabbit HPR-Polymer (RMR622G, Biocare Medical) for 30 min. The staining was visualized by the addition of the betazoid 3,3′diaminobenzidine chromogen kit solutions (BDB2004H, Biocare Medical). Finally, slides were counterstained with Mayer hematoxylin and observed through light microscopy (ApoTome). Negative controls were obtained by omitting the primary antibody on adjacent sections.

### Transmission electron microscopy

For lung ultrastructural analysis, glutaraldehyde-fixed fragments of lung tissue were processed and examined with a Philips Morgagni 268D transmission electron microscope (Philips, Brno, Czech Republic), as we previously described^54,55^.

### Statistical analysis

Results are expressed as mean ± SEM. Data were analyzed using ANOVA coupled with Tukey’s post hoc analysis. The statistical significance level was defined as *P* < 0.05. Data analysis was performed using GraphPad Prism (GraphPad Prism Software).

## Supplementary Information

Supplementary Information.

## Data Availability

All data are available in the main text and in supplemental materials; further inquiries can be directed to the corresponding author.
