# Supplementary material for: SARS-CoV-2 spike protein induces lung endothelial cell dysfunction and thrombo-inflammation depending on the C3a/C3a receptor signalling
Source: Sci Rep. 2023 Jul 14;13:11392. doi: 10.1038/s41598-023-38382-5 (PMC10349115; doi:10.1038/s41598-023-38382-5)
Supplement: Supplementary file 1 — Supplementary Information. [file 41598_2023_38382_MOESM1_ESM.pdf]

SARS-CoV-2 spike protein induces lung endothelial cell dysfunction and thrombo-inflammation depending on the C3a/C3a receptor signalling

Luca Perico[1†], Marina Morigi[1†], Anna Pezzotta[1], Monica Locatelli[1],
Barbara Imberti[1], Daniela Corna[1], Domenico Cerullo[1],
Ariela Benigni[1‡], and Giuseppe Remuzzi[1‡]

[1]Istituto di Ricerche Farmacologiche Mario Negri IRCCS, Bergamo, Italy

[†‡]These authors have contributed equally to this work and share first and last authorship, respectively

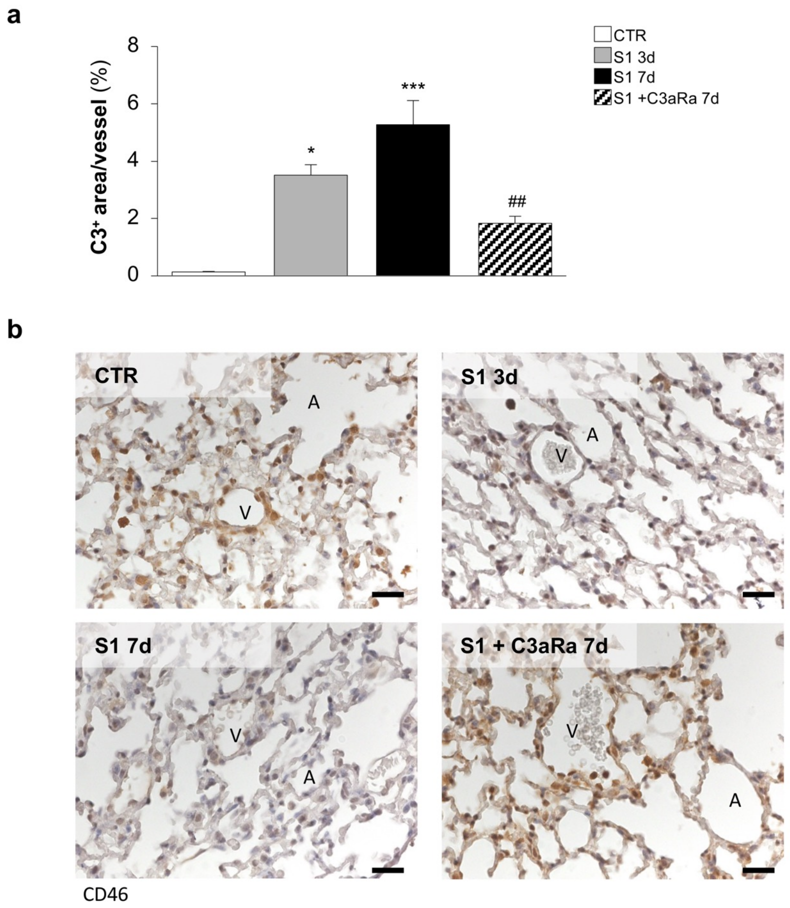

Supplementary Figure 1. C3aR antagonist inhibits C3 deposits and normalizes CD46 expression in lung tissue of hACE2-KI mice injected with S1. a Quantification of lung C3 deposits, expressed as C3 positive area *per* vessel, in CTR (n=4), S1-injected mice at 3d (n=3), and 7d treated or not with C3aR antagonist (C3aRa, n=6 *per* group). Results are presented as mean ± SEM. *P<0.05 and ***P<0.01 *vs* CTR; ##P<0.01 *vs* S1 at 7d. **b** Representative immunohistochemistry images of CD46 expression in the lung of CTR (n=4), S1-injected mice at 3d (n=3), and 7d treated or not with C3aRa (n=6 *per* group). Scale bars: 20 µm.

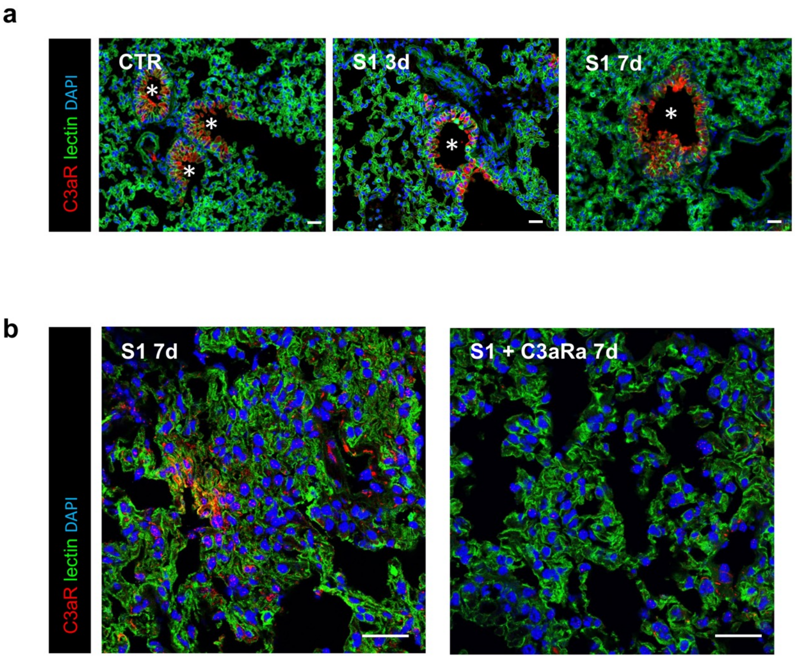

Supplementary Figure 2. C3aR expression in lung tissue of hACE2-KI mice. a Representative images of C3aR staining (red) in lung bronchiolar epithelial cells (asterisks) in CTR (n=4), S1-injected mice at 3 (n=3) and at 7d (n=6). Lung structures and nuclei are counterstained with WGA lectin (green) and DAPI (blu), respectively. Scale bars: 20 µm. **b** Representative images of C3aR staining (red) in lung tissue of S1-injected mice at 7d treated or not with C3aRa (n=6 *per* group). Lung structures and nuclei are counterstained with WGA lectin (green) and DAPI (blu), respectively. Scale bars: 20 µm.

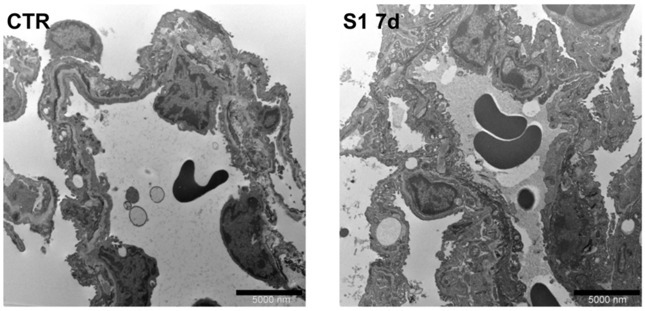

Supplementary Figure 3. Ultrastructural changes in lung arterioles of hACE2-KI mice injected with S1. Representative images of transmission electron microscopy micrographs of lung arterioles in CTR and S1-injected mice at 7d. Scale bars: 5000 nm.

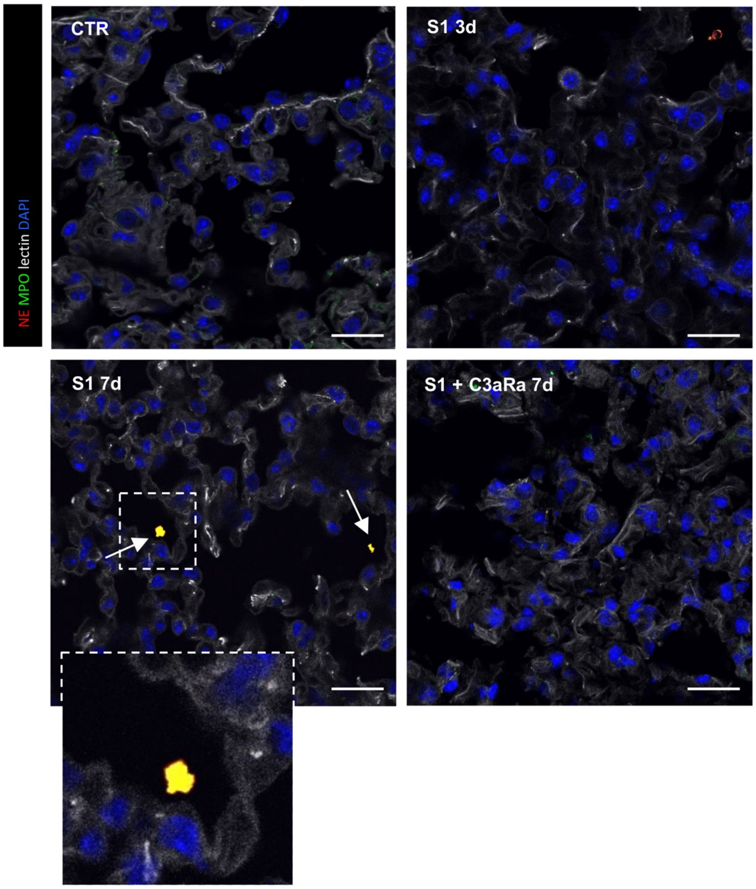

Supplementary Figure 4. C3aR antagonist limits NET formation in lung tissue of hACE2-KI mice injected with S1. Representative images of neutrophil elastase (NE, red) and myeloperoxidase (MPO, green) stainings in CTR (n=4), S1-injected mice at 3d (n=3), and 7d treated or not with C3aRa (n=6 *per* group). Scattered NET formation (arrows), identified by the co-staining (yellow) between NE and MPO, is evident only in mice treated with S1 at 7 days. Lung structures and nuclei are counterstained with WGA lectin (white) and DAPI (blu), respectively. Scale bars: 20 µm.
